# SASRT: Semantic-Aware Super-Resolution Transmission for Adaptive Video Streaming over Wireless Multimedia Sensor Networks

**DOI:** 10.3390/s19143121

**Published:** 2019-07-15

**Authors:** Jia Guo, Xiangyang Gong, Wendong Wang, Xirong Que, Jingyu Liu

**Affiliations:** State Key Laboratory of Networking and Switching Technology, Beijing University of Posts and Telecommunications, Beijing 100876, China

**Keywords:** video streaming optimization, semantic-aware, super-resolution, wireless multimedia sensor networks

## Abstract

There are few network resources in wireless multimedia sensor networks (WMSNs). Compressing media data can reduce the reliance of user’s Quality of Experience (QoE) on network resources. Existing video coding software, such as H.264 and H.265, focuses only on spatial and short-term information redundancy. However, video usually contains redundancy over a long period of time. Therefore, compressing video information redundancy with a long period of time without compromising the user experience and adaptive delivery is a challenge in WMSNs. In this paper, a semantic-aware super-resolution transmission for adaptive video streaming system (SASRT) for WMSNs is presented. In the SASRT, some deep learning algorithms are used to extract video semantic information and enrich the video quality. On the multimedia sensor, different bit-rate semantic information and video data are encoded and uploaded to user. Semantic information can also be identified on the user side, further reducing the amount of data that needs to be transferred. However, identifying semantic information on the user side may increase the computational cost of the user side. On the user side, video quality is enriched with super-resolution technologies. The major challenges faced by SASRT include where the semantic information is identified, how to choose the bit rates of semantic and video information, and how network resources should be allocated to video and semantic information. The optimization problem is formulated as a complexity-constrained nonlinear NP-hard problem. Three adaptive strategies and a heuristic algorithm are proposed to solve the optimization problem. Simulation results demonstrate that SASRT can compress video information redundancy with a long period of time effectively and enrich the user experience with limited network resources while simultaneously improving the utilization of these network resources.

## 1. Introduction

In recent years, wireless multimedia sensor networks (WMSNs), such as those used in transport management, health-care monitoring and live streaming services, have become increasingly popular. Users can watch videos taken by multimedia sensors everywhere at any time. The new generation of multimedia sensor nodes, such as DeepLens (a deep learning wireless camera released by Amazon), has superior hardware capabilities and can run deep learning algorithms. In WMSNs, there are few network resources due to multipath fading, shadowing and noise disturbance of wireless channels [1]. However, the quality and amount of video are gradually increasing over time. More network resources are needed to upload more multimedia data. When multiple multimedia services work simultaneously, network resources become seriously insufficient. In this situation, it is a major challenge to provide satisfactory multimedia services over WMSNs. Therefore, there is an increasing need for satisfactory multimedia service quality.

Compressing media data can reduce the user’s quality of experience (QoE) reliance on network resources. Existing video coding software, such as H.264 and H.265, focuses only on spatial and short-term information redundancy. However, videos usually contain redundancy over a long period of time. For example, in transport management, some objects (traffic police) and scenes (highways) are repeated. Therefore, compressing video information redundancy with a long period of time without compromising the user experience is a challenge.

In research on video coding, the general idea is to increase the compression efficiency of video coding [2]. However, these technologies still cannot compress long-term information redundancy. Most research on video transmission in WMSNs focuses on the optimization of route scheduling and adaptive streaming scheduling to improve the user’s video experience [1,3,4]. The above works enhance the user’s video experience by multi-path scheduling, adaptive transmission, etc. However, video quality and the amount of data transferred tend to be positively correlated. Under the limitation of network resources, it is difficult for the above transmission optimization technologies to enrich the objective quality of video.

In this paper, we have compressed long-term information redundancy through super-resolution technology and video transmission technology in WMSNs. It is feasible to compress video data by reducing data of the transmitted video and enriching the received video quality with super-resolution. Image super-resolution technology allows a higher-resolution image to be reconstructed from lower resolution during image processing. Super-resolution technologies have undergone significant development based on artificial intelligence [5]. Studies have found that obtaining semantic information about images can improve the performance of super-resolution technologies [6].

**Figure 1:**
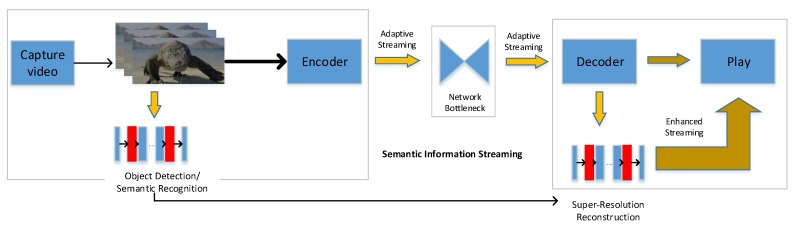
The idea of SASRT over WMSNs.

In this paper, we propose a semantic-aware super-resolution transmission for an adaptive video streaming system (SASRT) for WMSNs. The idea of SASRT is presented in Figure 1. The first step is being aware of semantic information in a multimedia sensor, and encoding video and semantic information. Semantic information of video can be obtained from object detection technologies such as Mask R-CNNs [7]. The identification location can also be the user side. The second step is enriching the quality of the received video. The received video is enriched by super-resolution assisted by semantic information on the user side. Specifically, the process is similar to the encoding of video data and decoding. The higher the efficiency of the super-resolution, the higher the proposed SASRT compression efficiency.

In contrast to applications in the field of image processing, more semantic information can be obtained from the original image in WMSNs. However, the identification of more detailed semantic information increases the consumption of network resources. Another method is utilized when limited semantic information is recognized on the user side. This method can reduce the consumption of network resources but increases the computational costs faced by the user side. Therefore, the major challenges faced by SASRT include where semantic information is identified, how to choose the bit rates of semantic and video information to encode, and how network resources are allocated to video and semantic information.

The above challenges are solved in the SASRT framework. In addition, in contrast to traditional transmission methods, the objective of SASRT is to encode and transmit different bit-rate video and semantic information. Then, we introduce a novel mathematical model for characterizing the user experience to be affected by certain factors in the proposed SASRT. Based on the model, the problem of maximum income is considered. Then, the problem is proven to be an NP-hard problem. Finally, three adaptive strategies and a heuristic algorithm are proposed to solve the NP-hard problem. The SASRT scheme can be used in the scene of smart cities [8,9,10,11].

The remainder of this paper is organized as follows. In Section 2, we review research on the video transmission and super-resolution technologies. In Section 3, the system architecture of SASRT, the model and some analyses are described. Then, a heuristic algorithm is presented to address the above issues. In Section 4, we build a real transmission system and illustrate the performance evaluation for verifying our method. In Section 5, we conclude our work.

## 2. Related Work

In this section, we review research on video coding, video transmission and super-resolution technologies.

Studies on video coding are based primarily on reliable systems. Regardless of the compression algorithm, the same data are required before and after compression. A new image-coding scheme that uses a region-adaptive prediction method with modified current specifications on JPEG XT Part 7 is proposed in [12]. In [2], a video analysis and coding framework is proposed to jointly compress near-identical videos. Two adaptive encoding techniques are proposed to reduce the bit rate of omnidirectional videos after compression in [13]. In [14], a fast quad-tree structure determination scheme for encoding depth videos in 3D high-efficiency video coding is proposed. That paper focuses on video data compression during transmission. Moreover, advanced video coding technologies are applicable to our proposed architecture.

Many scholars have performed research on video transmission in WMSNs. In [3], a power-efficient and multi-path video packet scheduling algorithm is proposed to improve the user’s QoE. In [4], the authors presented an adaptive cross-layer framework for transmitting multimedia content over WMSNs. The authors of [1] presented an energy-aware and adaptive cross-layer scheme for video transmission over wireless sensor networks, which is an extension of the previously introduced scheme in [4]. The idea of video adaptive transmission is to provide users with appropriate media content by detecting the available bandwidth, client buffer and CPU capacity in real time [15]. In [16], an optimization technology for video transmission over mobile cloud networks is proposed. In [17], a new architecture is proposed to address the problem of reduced video transmission capacity resulting from TCP slow start. A scheme for adaptively transmitting omnidirectional video is proposed in [18] and greatly improves the transmission efficiency. In [19], a quantum-assisted multi-user detection (QMUD) in a multi-user system is proposed to improve performance and reduce complexity. The authors of [20] investigated a resource allocation approach in wireless networks under total bandwidth and energy efficiency constraints. A joint optimization of the bandwidth allocation and power control is proposed in that paper. The above work enhances the user’s video experience by reducing latency, stagnation, jitter, etc. However, video quality and the amount of data transferred tend to be positively correlated. When it is not possible to transmit video data with the lowest quality level, the above studies cannot provide users with satisfactory video services under insufficient network resources.

Many papers have studied super-resolution technologies. Two types of methods are used for super-resolution imagery. The first type is the use of traditional mathematical interpolation methods to achieve super-resolution images (e.g., [21,22,23]). The other type focuses on achieving super-resolution images using artificial intelligence. The development of artificial intelligence has had a revolutionary impact on super-resolution technologies. The super-resolution convolutional neural network (SRCNN) has been proposed to use neural networks for achieving super-resolution images [24]. However, the SRCNN has few convolutional layers. In [25], more convolutional layers are proposed in a deeply recursive convolutional network (DRCN). The efficient subpixel convolutional neural network (ESPCN), which calculates convolutions directly on low-resolution images and obtains high-resolution images, is proposed in [26]. The authors proposed a method to use time-series images in video for super-resolution reconstruction. In [27], an algorithm for high dynamic range (HDR) and super-resolution imaging from a single image is presented. In [28], a multitask learning framework is developed to jointly train dual-stream edge-driven encoder–decoder networks, which combine an edge stream-based encoder–decoder network and a color stream-based encoder–decoder network. Moreover, a total loss function combining edge loss and color loss is presented to achieve an optimal tradeoff between image fidelity and texture details. Recently, the generative adversarial network has been used for super-resolution reconstruction in [5]. In [6], the authors proposed a new method that makes the super-resolution image texture more natural. In [29], a super-resolution video method named the enhanced video super-resolution network with residual blocks is proposed. A motion compensation process is not needed for the super-resolution video method.

Some scholars are also working on topics related to deep learning and video (e.g., [30,31]). In [30], the authors mainly studied a scheme for edge video analysis based on deep learning. In [31], the authors studied a super-resolution technology for video. However, this type of research work is simply a study of video clarity enhancement algorithms. The algorithm improves the super-resolution video efficiency based on video characteristics such as timing. In contrast to the focus of our research, the above studies examine how to increase the resolution of the low-resolution video that is received after transmission. They do not consider some information about the high resolution before the video transmission and restrictions in the network.

In summary, coding technology and network transmission technology have been extensively developed. However, video data will increasingly dominate mobile network traffic. It is predicted that, by 2022, 79 percent of mobile data traffic will be video, up from 59 percent in 2017 [32]. Video transmission optimization methods can be divided into certain main categories. The first category attempts to reduce the quality of the transmitted video, reducing delay, jitter and re-buffering. The second category attempts to improve the network transmission efficiency using predictive technology and increasing inter-network caching. The third category attempts to enhance the user experience through resource balancing. However, given network resource constraints, there is a theoretical upper limit on the optimization results of the above methods. Increasing the coding efficiency of the video is one possible solution to the above problems. However, existing video coding software, such as H.264 and H.265, focuses only on spatial and short-term information redundancy. However, videos usually contain redundancy over a long period of time. Reducing long-term video redundancy is thus the goal of this paper. Our research focuses on using useful information identified from high-resolution videos before upload and real-time network status to improve super-resolution performance. This approach can achieve our optimization goals: compressing long-term video redundancy during upload, thereby reducing network resource consumption without compromising the user experience.

## 3. System Architecture, Model and Analysis

In this section, we introduce the concept of SASRT over WMSNs and describe the system architecture and model. Then, three adaptive strategies are proposed and analyzed individually.

### 3.1. System Architecture

**Figure 2:**
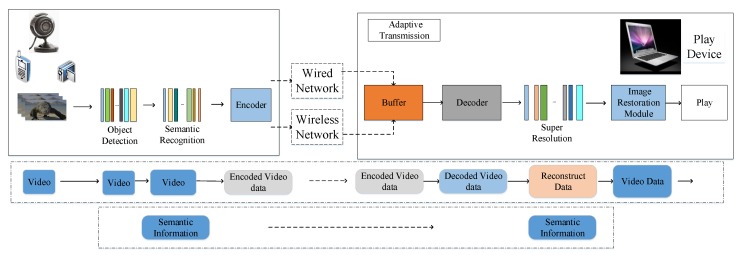
The proposed SASRT architecture for adaptive transmission over WMSNs.

Figure 2 shows the system architecture of SASRT. The system framework of SASRT consists of multimedia sensor (adaptive video encoder), the Internet, and users. Artificial intelligence algorithms are run on both the multimedia sensor and the client. On the multimedia sensor, a scene recognition algorithm and a semantic recognition algorithm identify the scene and semantics of the video, respectively. Semantic recognition can be achieved on the multimedia sensor or on the user side. Note that three adaptive strategies proposed in this section can be run on the multimedia sensor or on a separate intermediate proxy server.

SFTGAN is one of the best super-resolution technologies, as presented in [6]. More visually pleasing performances, especially in terms of realism and textures, are obtained by SFTGAN. In this article, SFTGAN is used and adapted to video upload. In that paper, more semantic information can be obtained, which is beneficial for super-resolution reconstruction.

Note that our method does not depend on SFTGAN. For other super-resolution methods, the gain in super-resolution performance achieved through semantic information is different. Thus, our method is still effective.

**Figure 3:**
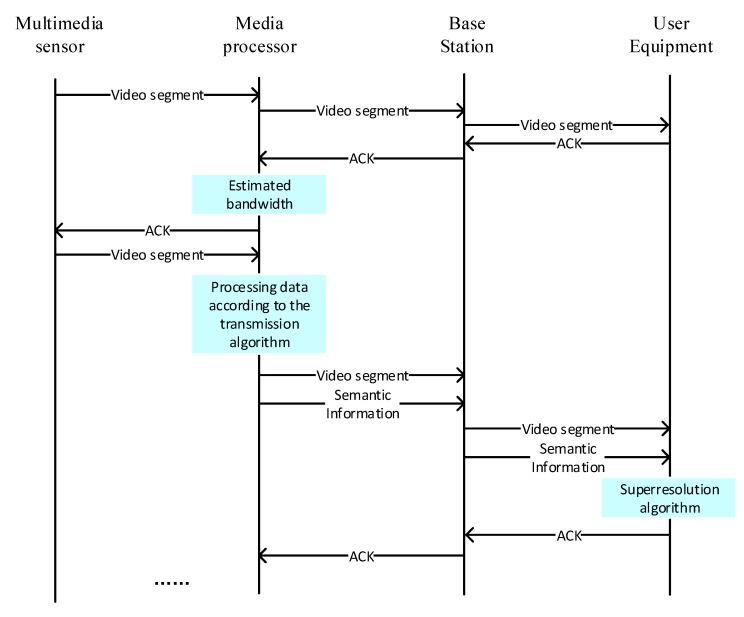
Process flow diagram of the proposed SASRT architecture for adaptive transmission over WMSNs.

The transmission process of SASRT is shown in Figure 3. The media processor can be located in a multimedia sensor, such as the deep learning wireless camera released by Amazon or an edge computing node. The TCP protocol is used during transmission. First, the multimedia sensor captures the video and transmits the lowest quality video to the media processor when the media sensor receives the video data for the first time, and it is forwarded directly to the user through the base station. Then, the user receives the data and returns a confirmation message. The media processor estimates the network bandwidth at the next moment based on the confirmation message. Third, the media processor processes the received video data according to the estimated information, such as through coding, scene recognition and semantic information extraction. Video data and semantic information are simultaneously sent to the user. Finally, the super-resolution algorithm is used assisted by the video data and semantic data at the user’s equipment.

### 3.2. System Model

In this section, we describe the system model and formulate the optimization problem.

There are many multimedia sensor application scenarios in daily life such as health-care monitoring and live streaming. The application of different super-resolution models for different multimedia sensor scenarios can improve the efficiency of super-resolution technologies. Assume that a scenario for a multimedia sensor is $V^{a}$, where $V^{a}\in \left[V^{1},V^{2},\ldots ,V^{c}\right]$, and *E* is used to express the user experience.

(1) $$E=\sum_{i=1}^{s}E_{i},$$

where *s* is the number of video segments. $E_{i}$ depends on the video quality function $Q(S_{i})$ and the negative gain function $RJ^{i}$, and $Q(S_{i})$ is a pre-estimated utility-rate function for each video program, where the utility can be a subjective evaluation metric (such as MOS) or an objective evaluation metric (such as PSNR or SSIM) [33].

(2) $$E_{i}=Q\left(S_{i}\right)-\xi {RJ}^{i},$$

where ${RJ}^{i}$ is the computational cost of semantic recognition and the additional bandwidth consumption caused by the semantic information in this paper. Therefore, ${RJ}^{i}=\chi_{1}cost_{c}+\chi_{2}cost_{b}$, and $cost_{c}$ is the cost of the bandwidth consumption when semantic recognition is performed on the multimedia sensor. $cost_{b}$ is the computational cost of semantic recognition when semantic recognition is performed on the client side. $\chi_{1}$ and $\chi_{2}$ represent the weights of the different costs. ξ is used to control the trade-off between video quality and negative gain.

(3) $$\left\{\begin{array}{c} S_{i}=\left\{{f_{i}}^{1},{f_{i}}^{2},\ldots ,{f_{i}}^{S_{t}*S_{f}}\right\}\\ CNN\left({f_{i}}^{j}\right)=\left\{{se_{1}}^{j},{se_{2}}^{j},\ldots ,{se_{k}}^{j}\right\} \end{array}\right.k\geq 1,$$

where $S_{i}$ is expressed as the *i*th segment. Assume that each video segment plays for $S_{t}$ seconds. The frame rate of the video is $S_{f}$ fps. Therefore, there are $S_{t}*S_{f}$ frames in a video segment. CNN expresses a semantic recognition deep learning algorithm such as Mask R-CNN. ${f_{i}}^{j}$ is expressed as the *j*th frame in the *i*th segment. In the formulas above, the value of $Q(S_{i})$ is very important and can be calculated by

(4) $$Q\left(S_{i}\right)=Q\left({f_{i}}^{1}\right)+Q\left({f_{i}}^{2}\right)+\ldots +Q\left({f_{i}}^{S_{t}*S_{f}}\right),$$

(5) $$Q\left({f_{i}}^{j}\right)={ℜ}_{V^{a}}\left(b_{\gamma }\left({f_{i}}^{j}\right)\right),$$

where ${ℜ}_{V^{a}}\left(b_{\gamma }\left({f_{i}}^{j}\right)\right)$ is a function of the relationship between video quality and video bit rate in the video scenario $V^{a}$. In other words, the function ${ℜ}_{V^{a}}$ expresses the process of super-resolution reconstruction, and the model for the super-resolution reconstruction is determined by the video scenario $V^{a}$. $b_{\gamma }$ is the bit rate of the video.

(6) $$Q\left(S_{i}\right)=\sum_{j=1}^{S_{t}*S_{f}}{ℜ}_{V^{a}}\left(b_{\gamma }\left({f_{i}}^{j}\right),b_{\gamma }\left({se_{k}}^{j}\right)\right),$$

(7) $$b_{\gamma }\left({se_{k}}^{j}\right)=\sum_{a=1}^{k}{{b_{\gamma }}^{*}}_{se_{a}},$$

where ${se_{k}}^{j}$ is expressed as the semantic information of the quality level *k* in the *j*th frame. $b_{\gamma }\left({se_{k}}^{j}\right)$ is expressed as the bit rate of the *k*th piece of semantic information. ${{b_{\gamma }}^{*}}_{se_{1}}$ is the bit rate of the first quality-level semantic information. ${{b_{\gamma }}^{*}}_{se_{c}},1<c\leq k$ represents the difference between the semantic information bit rate of quality level *C* and the semantic information bit rate of quality level C−1. $C_{x}$ is a Boolean function and is used to control the selection of the semantic information quality level. The value of $C_{x}$ can be 0 or 1.

(8) $$C_{x}\in \left\{C_{1},C_{2},\ldots ,C_{\varsigma }\right\},1\leq x\leq \varsigma ,$$

(9) $$\left\{\begin{array}{c} b_{\gamma }\left({f_{i}}^{j}\right)=ℑ\left(\left\{C_{1},C_{2},\ldots ,C_{\varsigma }\right\}\cdot \left\{{l_{1}}^{j},{l_{2}}^{j},\ldots ,{l_{\varsigma }}^{j}\right\}\right)\\ \sum_{e=1}^{\varsigma }C_{e}=1\\ b_{\gamma }\left({f_{i}}^{j}\right)+b_{\gamma }\left({se_{k}}^{j}\right)\leq B \end{array}\right.,$$

where *ℑ* is a function of the relationship between the video bit rate and the video quality level, *l* is the video frame level, ς is the number of video quality levels stratified, $bit_{se_{j}}$ is the bit rate of the semantic information $se_{j}$, and *B* is the bandwidth at the current time. The selection of $\left\{C_{1},C_{2},\ldots ,C_{\varsigma }\right\}$ is related to the network bandwidth *B*, which can be estimated by

(10) $$B=\left\{\begin{array}{cc} \frac{b_{\mu }\times t_{s}}{t_{\mu }-t_{\mu -1}}\;\mathrm{the}\;\mathrm{first}\;\mathrm{segment}\\ \delta \times \frac{(b_{\mu -1}\times t_{s})}{t_{\mu -1}-t_{\mu -2}}+\left(1-\delta \right)\times B^{*} & \mathrm{otherwise} \end{array}\right.,$$

where *B* is the predicted network bandwidth, $b_{\mu }$ is the bit rate of the μth segment of the video, $t_{\mu }$ is the received time, $t_{\mu -1}$ is the sent time, $t_{s}$ is the playback time of the segment, $B^{*}$ is the available bandwidth for the last segment, and δ is the weight given to the current bandwidth. Using other methods for estimating the available bandwidth, our model remains feasible.

No super-resolution model is perfect; different models achieve different super-resolution reconstruction efficiencies. For example, a human image super-resolution model used to improve the resolution of an animal image will be less effective. Therefore, it is necessary to use different super-resolution models to improve the resolution of different types of images. The collection $\left\{\ell_{1},\ell_{2},\ldots ,\ell_{c}\right\}$ is used to represent different super-resolution models.

(11) $$\left\{\begin{array}{c} \mathrm{maximize}(Q\left({f_{i}}^{j}\right))\\ Q\left(S_{i}\right)=\left\{{ℜ}_{\ell_{1}}\left(V^{1}\right),{ℜ}_{\ell_{2}}\left(V^{2}\right),\ldots ,{ℜ}_{\ell_{c}}\left(V^{c}\right)\right\}.\\ \left\{C_{1},C_{2},\ldots ,C_{c}\right\} \end{array}\right..$$

Assuming the traditional method under the above conditions, the bit rate of the video is $bit_{com}$, which can be calculated by

(12) $$bit_{com}=\max\left\{b_{\gamma }\left(l\right)|l=1..\varsigma ,b_{\gamma }\left(l\right)\leq B\right\}.$$

In its most generic form, the problem can be formulated as follows.

(13) $$\begin{array}{c} \;\mathrm{maximize}(bit_{com}-\left(b_{\gamma }\left({f_{i}}^{j}\right)+b_{\gamma }\left({se_{k}}^{j}\right)\right))\\ \mathrm{subject}\;\mathrm{to}\;(1)–(11),E(b_{\gamma }\left({f_{i}}^{j}\right)+b_{\gamma }\left({se_{k}}^{j}\right))\to ||>\\ E(bit_{com}) \end{array}.$$

### 3.3. Complexity Analysis

We first find a special scene of the problem given by Equation (13). Then, we prove that it is equivalent to a well-known NP-hard problem, which is the classic exact-cover problem. Finally, Equation (13) is an NP-hard problem to prove.

**Theorem** **1.** *The problem defined by Equation (13) is an NP-hard problem.*

**Proof.**  Consider a special case of Equation (13) with $\ell_{1}=1,\ell_{2}=0,\ldots ,\ell_{k}=0$, $E(b_{\gamma }\left({f_{i}}^{j}\right)+b_{\gamma }\left({se_{k}}^{j}\right)=E\left(bit_{com}\right)$. Equation (13) reduces to

(14) $$\begin{array}{c} \;\mathrm{maximize}(bit_{com}-\left(b_{\gamma }\left({f_{i}}^{j}\right)+bit_{se_{j}}\right))\\ \mathrm{subject}\;\mathrm{to}\;\sum_{o=1}^{k}{b_{\gamma }}^{*}\left({se}_{o}\right)\leq B-b_{\gamma }\left({f_{i}}^{j}\right) \end{array},$$

where $B-b_{\gamma }\left({f_{i}}^{j}\right)$ is the available bandwidth allocated for the semantic information se. We transform the problem of Equation (14) into a set of problems.

We represent all sets in *S* by bit vectors of length $3k^{\prime }$. For example, $\left\{{se}_{1},{se}_{2},{se}_{6}\right\}$ and $\left\{{se}_{4},{se}_{5},{se}_{6}\right\}$ are represented by 110001 and 000111, respectively. Each subset satisfies the constraints of *S* as a row composed of a matrix *A*. The integer corresponding to each set can be written in the base-(k+1) system. The value of each collection can be calculated by $V=\sum_{o=0}^{{3k}^{\prime }-1}{\left(m+1\right)}^{o}$.

Suppose that our problem has an extreme value; then, we construct the integers $b_{\gamma }\left({se}_{1}\right),b_{\gamma }\left({se}_{2}\right),\ldots ,b_{\gamma }\left({se}_{k}\right)$, and $B-b_{\gamma }\left({f_{i}}^{j}\right)$. The value of $B-b_{\gamma }\left({f_{i}}^{j}\right)$ corresponds to the sequence {1,1,…1} in the base-(k+1) system. Therefore, our problem is finding a set of *V* with sum $B-b_{\gamma }\left({f_{i}}^{j}\right)$. Another way of representing this is finding a set of rows in the matrix *A*, where each column in the set contains exactly a single 1.

A classic exact-cover problem is the following: given a matrix *B* consisting of 0 s and 1 s, the exact cover problem is to find a set of rows such that each column in the set contains exactly a single 1. Therefore, the exact covering problem is equivalent to the special case of Equation (13).

This completes the proof.  □

It follows that the problem defined in Equation (13) is an NP-hard problem. The globally optimal solution is difficult to find. In general, an approximate solution is usually obtained by a heuristic algorithm. In this paper, a heuristic algorithm is used to solve the problem.

### 3.4. Method

Our implementation is as follows: SFTGAN is used to identify the semantic information.

The following are the three adaptive strategies used during video upload. In practice, these three adaptive strategies require overall consideration to improve the efficiency of the super-resolution reconstruction.

**Adaptive video semantic encoding strategy**:

Our goal is to reduce the uploading of video data without compromising the user experience. Semantic recognition can improve the compression efficiency of SFTGAN without compromising the user experience. We propose an adaptive semantic information encoding strategy that represents a trade-off between different quality levels of semantic information.

We assume that on the multimedia sensor, the computational cost of semantic recognition applied to a video frame is cost. Many factors affect semantic recognition. Semantic information can be encoded with different quality levels. The computational cost of semantic information differs at different quality levels. Therefore, the total cost of processing a video segment $S_{i}$ is

(15) $$Tcost_{i}=\sum_{\phi =1}^{\omega }{cost_{i}}^{\phi },1\leq \omega \leq S_{t}*S_{f},$$

where ω is the number of frames that require semantic recognition. Moreover, the video quality can be estimated according to experiments in which different bit-rate semantic information is recognized.

(16) $$\left\{\begin{array}{c} \mathrm{maximize}(Q^{{}^{\prime }}\left({f_{i}}^{j}\right)-\xi {cost_{i}}^{j})\\ \sum_{j=1}^{S_{t}*S_{f}}\left({b_{\gamma }}_{j}+{b_{\gamma }}_{se_{j}}\right)\leq B \end{array}\right.,$$

Moreover, not all image frames in the video need to be semantically recognized. The differences between adjacent frames of a video are small. Therefore, the frame semantic recognition frequency of different video scenarios also varies. For different video scenes, the frame frequency of semantic recognition can be obtained experimentally or empirically.

**Adaptive semantic recognition location selection strategy**:

In this paper, the image semantic information of an image can be calculated on a multimedia sensor or on the user side. The trade-off between computational cost and bandwidth consumption caused by the different semantic recognition locations is important. Assume that the video semantic se has been selected and that the video quality can be estimated. First, we define two Boolean variables, $C_{\varpi }$ and $C_{\varrho }$.

(17) $$\left\{\begin{array}{c} C_{\varpi }=\left\{0,1\right\}\\ C_{\varrho }=\left\{0,1\right\}\\ |C_{\varpi }-C_{\varrho }|\neq 0 \end{array}\right..$$

Second, the semantic recognition location can be selected by

(18) $$\left\{\begin{array}{c} \mathrm{maximize}(bit_{com}-\left(b_{\gamma }\left({f_{i}}^{j}\right)+bit_{se_{j}}\right))\\ E_{se}=E_{se}\left(i\right)-C_{\varpi }\times \chi_{1}cost_{c}-C_{\varrho }\times \chi_{2}cost_{b} \end{array}\right.,$$

where $cost_{c}$ is the computational cost of semantic recognition performed on the user side. The $cost_{b}$ is the cost of the bandwidth consumption when semantic recognition is performed on the multimedia sensor. $\chi_{1}$ and $\chi_{2}$ represent the weights of the different costs.

**Adaptive bit-rate encoding strategy**:

The selection of the bit rate depends on many parameters, such as the cache and bandwidth. In this paper, we consider only the bandwidth as the sole determinant during bit-rate selection. There are many bandwidth prediction techniques. However, bandwidth prediction is not the focus of our research. Therefore, the bandwidth can be calculated by Equation (10). Moreover, the bit rate of the video can be calculated by

(19) $$\left\{\begin{array}{c} \mathrm{maximize}(bit_{com}-\left(b_{\gamma }\left({f_{i}}^{j}\right)+bit_{se_{j}}\right))\\ \sum_{j=1}^{S_{t}*S_{f}}\left(b_{\gamma }\left({f_{i}}^{j}\right)+bit_{se_{j}}\right)\leq B \end{array}\right.,$$

where $b_{\gamma }\left({f_{i}}^{j}\right)$ is the bit rate of the *j*th frame. Based on the first adaptive strategy, our goal is to maximize the compression efficiency.

### 3.5. Proposed Solution

This article includes three adaptive strategies that influence each other. We propose a heuristic algorithm to solve the optimization problem with the above strategies. The basic idea of the proposed algorithm is as follows:

Our algorithm is divided into four phases. In the first phase, the semantic recognition location can be calculated according to Equation (18). In the second phase, the semantic information bit rate and video bit rate are selected for encoding. According to the above description, the semantic information and video information of different levels are completely arranged, and the video experience of the user is estimated in different situations. In the three phases, we define a utility function, denoted UtilitySlope, as the ratio of the sum of the utility gain and the sum of the required bandwidth, as shown in Equation (20). Our goal is to maximize UtilitySlope at every step of the algorithm. *b* is the bit rate of the video with different semantics. A larger UtilitySlope indicates a greater utility gain of the average bandwidth, which means that the allocation strategy is more efficient. In this phase, the utility function UtilitySlope of each video with different levels, which have different bit rates $b_{\gamma }\left(l\right)$, is estimated first. Then, we rank the values of UtilitySlope in order of decreasing importance. According to the values of UtilitySlope, which are arranged from high to low, the bit rate of the video is determined until the network resources are no longer sufficient.

(20) $$UtilitySlope\left(j\right)=\frac{bit_{com}-\left(b_{\gamma }\left({f_{i}}^{j}\right)+bit_{se_{j}}\right)}{b}.$$

In the last phase, the video information is reconstructed using matched super-resolution models. If there is no matching super-resolution model, $\mathrm{maximize}({ℜ}_{\ell })$ will be found from the predicted values. The pseudocode of this algorithm is shown in Algorithm 1.

**Algorithm 1:** Transmission strategy algorithm. **Require:**   Initialize video frame {$f^{1},f^{2},\ldots ,f^{S_{t}*S_{f}}$}.   Initialize cost.   Identify the scenario of video frame {$f^{1},f^{2},\ldots ,f^{S_{t}*S_{f}}$}.   Identify the semantic recognition cost cost.   Assume that the video semantics of a video frame can encode   {$se_{1},se_{2},\ldots ,se_{k}$}. Initialize the cost of bandwidth consumption $cost_{b}$   Calculate the location of semantic recognition by Equation (18). 1:**if** semantic recognition is performed on the multimedia sensor. **then**2: $cost_{b}=0$.3:**else**4: $cost_{b}\neq 0$.  5:**end if**  6:**for all** {$se_{1},se_{2},\ldots ,se_{ks}$} **do**7:  **for all** {$l_{1},l_{2},\ldots ,l_{\varsigma }$} **do**8:   Estimate the user’s video experience, $E_{i}$.  9:  **end for**  10:**end for**Initialize bandwidth *B*.The image quality level of each frame is {$l_{1},l_{2},\ldots ,l_{\varsigma }$}.The bit rate corresponding to each quality level is {$b_{\gamma }\left(l_{1}\right),b_{\gamma }\left(l_{2}\right),\ldots ,b_{\gamma }\left(l_{\varsigma }\right)$}  11:**for all** {$b_{\gamma }\left(l_{1}\right),b_{\gamma }\left(l_{2}\right),\ldots ,b_{\gamma }\left(l_{\varsigma }\right)$} **do**12:  **for all** {$se_{1},se_{2},\ldots ,se_{ks}$} **do**13:   Calculate the utility function UtilitySlope of all video quality   levels for each frame.  14:  **end for**  15:**end for**Rank the values of UtilitySlope in decreasing order of significance.According to the values of UtilitySlope, which are arranged fromhigh to low, select the value of $b_{\gamma }\left(l\right)$.Initialize the super-resolution model $\left\{\ell_{1},\ell_{2},\ldots ,\ell \right\}$.Select the super-resolution model for each video semantic.  16:**for all**$\left\{V^{1},V^{2},\ldots ,V^{a}\right\}$**do**17: **if** Video scene $V^{x}$ has a super-resolution model *ℓ* that matches it **then**18:  Select the super-resolution model *ℓ* for the video scene $V^{x}$.  19: **else**20:  Find $\mathrm{maximize}({ℜ}_{\ell })$ from the predicted value.  21: **end if** 22:**end for**


## 4. Performance Evaluation

### 4.1. Experimental Method

**Figure 4:**
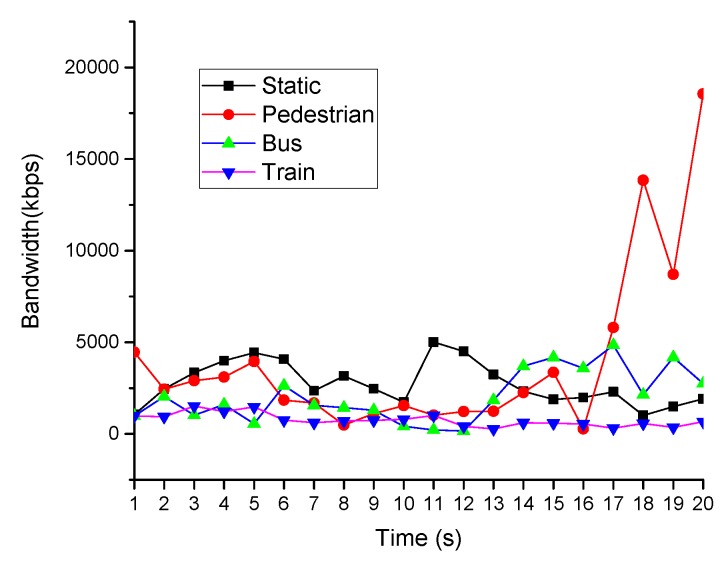
Network bandwidth.

We experimented on our solution by building an actual transmission system. To repeat the experiments, we used a PC with the Ubuntu 16.04 system emulation multimedia sensor. The client was connected to the multimedia sensor through a wireless cellular network. In the experimental environment, we considered only the impact of the bandwidth on the transmission performance. In our experiment, we assumed that the user is mobile. Therefore, we used the bandwidth change data collected as in [34]. Link loss rate was set to 2% and delay was set to 2 ms. A script was used to control the network bandwidth in four states at different motion speeds: static, pedestrian, bus and train (Figure 4).

Current popular super-resolution methods are divided into two categories: GAN-based methods and PSNR-oriented methods. The first type of method consists of SFTGAN, SRGAN and EnhanceNet [35]. The other methods include SRCNN, VDSR [36] and LapSRN [37]. The evaluation of GAN-based methods are not applied to existing objective evaluation methods such as SSIM and PSNR [6]. To evaluate the performance of our system, the method in [6] was used. In the experimental results, we intercepted certain frames in the video at the same time for comparison.

User experience is defined by Equation (1). The parameter σ was referenced by Guo et al. [33]. In this paper, σ consists of two parameters: $\chi_{1}$ and $\chi_{2}$. Taking the trade-off between the average video quality and cost into consideration in our paper, we used $\chi_{1}=0.1$ and $\chi_{2}=0$ as the parameter values. The multimedia sensor cost is positively related to the amount of processed data [38]. For the video quality, we used subjective measurement methods in this experiment.

Two 1080P sequence videos, taken from the video “Planet Earth”, were used to simulate the video captured by the multimedia sensor. The “Planet Earth” video is a documentary made by the British Broadcasting Corporation. The video sequences include 250 frames and are repeated. The frame rate is 25 frames per second, and the GoP size is 8 images. A total of 10 quality levels can be encoded. There is similarity between adjacent video frames. Therefore, the semantic information about the video is detected every 25 frames.

The SASRT metrics for the analysis described here are efficiency and throughput, defined as follows:

- Efficiency: A subjective quality assessment method was used. The same video frames under different strategies were compared.
- Throughput: The amount of data successfully transmitted in a unit of time. The greater is the throughput, the larger is the amount of data transmitted per unit time.
- Playback Stability: We measured the video playback instability with the following formula:
  (21) $$PS=1-\frac{\sum_{f=0}^{S-1}\left(|l_{h}-j_{S-f}^{{}^{\prime }}\right|*\omega \left(d\right))}{\sum_{f=0}^{S-1}(l_{h}*\omega \left(d\right))},$$
  where PS is the stability index, which is 1 minus the weighted sum of all switch steps in the previous segments divided by the weighted sum of the highest received quality value during transmission time. *S* is the number of segments. $l_{h}$ represents the highest received quality value of the transmitted video during transmission time. $j_{S-f}^{{}^{\prime }}$ represents the received quality of the (S−f)th video segment. ω(d) = S−f assigns higher penalties to more recent quality switches. If video rebuffering occurs, the current video segment quality is zero. The closer the value of PS is to 1, the better is the stability.

We conducted three separate experiments in the same network environment. The first method was the traditional DASH transmission method in [38]. The second method was where the semantic recognition location of the former method was calculated at the multimedia sensor. The third method was the proposed Algorithm 1.

Referring to the international standard ITU-R BT.1788, 20 people with normal vision were chosen for the subjective quality assessment. Videos were viewed by the user at the same distance. The received video was saved and played separately for different groups of users. The video was paused every second, and the users performed a subjective scoring every 1 s. They gave their scores based on subjective feelings. The score ranged from 1 to 5, with 1 indicating the lowest quality level and 5 indicating the highest quality level. The average score for all users was used as the final score.

We also calculated the PSNR and SSIM values of the received image. To calculate the PSNR and the value of SSIM, we used the bilinear interpolation algorithm to make the resolution of the video received by the first method equal to the resolution of the original highest quality video. Because the video resolution received by the latter two methods was already equal to the resolution of the highest quality video before uploading, no processing was required.

A no-reference quality evaluation method based on visual perception is used in [39]. A larger value means better quality. The super resolution of the video will produce some redundant information compared to the original video. Therefore, a blind quality evaluation method was more suitable for this study.

### 4.2. Experimental Result

In this paper, A1 and B1 represent videos A and B, respectively, transmitted by the first method. A2 and B2 represent videos A and B, respectively, transmitted by the second method. A3 and B3 represent videos A and B, respectively, transmitted by the third method.

**Figure 5:**
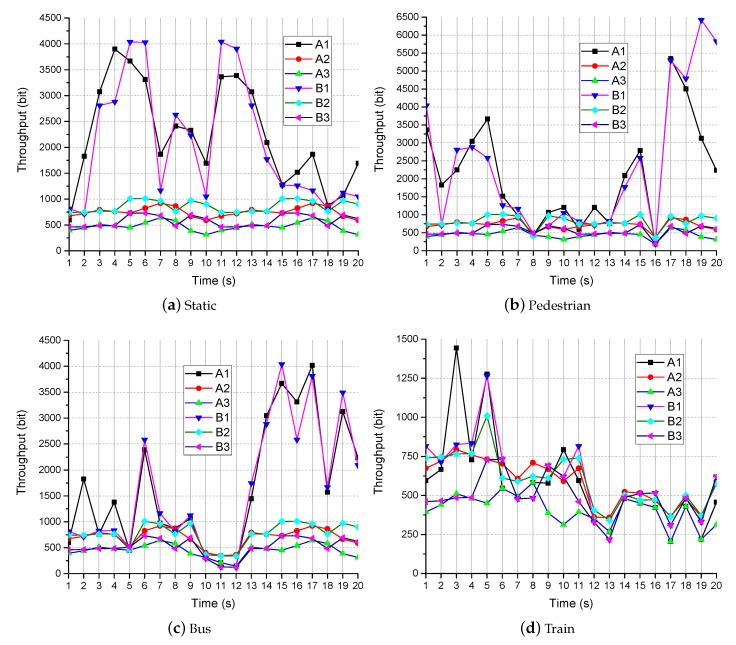
The throughput in different network environments.

The throughputs of the three transmission methods are shown in Figure 5. Figure 5a shows the throughput when the user is static. Our approach (A3 and B3) saves substantial bandwidth while maintaining video quality. When the semantic recognition location was calculated on the multimedia sensor (A2 and B2), SASRT can save an average of 60 percent of network resources. SASRT (A2, A3, B2, and B3) can save an average of 70 percent of network resources. Figure 5b–d shows the throughputs when the user is moving. Therefore, the bandwidth may change significantly with the user’s location. The average bandwidth consumption of TSA (A3 and B3) is also substantially smaller than the other methods in Figure 5b,c. Figure 5d shows the throughput when the user is moving at high speed. The bandwidth of the network is often limited and is sometimes zero. We do not consider the case in which the bandwidth is zero. The throughputs of all methods (A1, A2, A3, B1, B2, and B3) are not significantly different in Figure 5d because lower-resolution video data are not encoded on the multimedia sensor. However, SASRT (A2, A3, B2, and B3) can provide users with videos of better quality.

**Figure 6:**
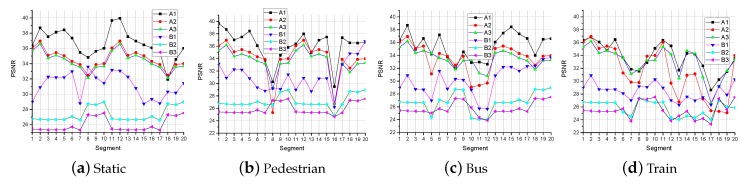
The PSNR in different network environments.

**Figure 7:**
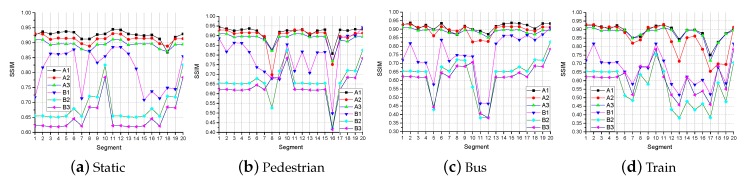
The SSIM in different network environments.

The results of PSNR and SSIM are shown in Figure 6 and Figure 7. Figure 6 and Figure 7 shows that, for Video A, the PSNR and SSIM of video received by all methods (A1, A2, A3, B1, B2, and B3) are not much different. However, the above results are not applicable in Video B. The PSNR and the SSIM of the video received by the latter two methods (A2, A3, B2, and B3) do not change much under the four network states. However, when the network changes greatly, the PSNR and SSIM of video received by Method 1 (A1 and B1) have a large change. In the case of a poor network environment, the PSNR and SSIM of video received by Method 1 (A1 and B1) are more similar to received by other methods (A2, A3, B2, and B3). The reasons are as follows: the super-resolution algorithm SFTGAN we used is a GAN-based method. This method has acquired a large number of parameters after a lot of confrontation learning. In the process of super-resolution reconstruction, a large amount of information for improving the user’s subjective feeling is added for low-resolution video. However, from a computer perspective, the video processed by SFTGAN is very different from the original HD video. The PSNR and SSIM of the video obtained by the first method (A1 and B1) are higher than the latter two methods (A2, A3, B2, and B3). This is the reason that the latter two methods (A2, A3, B2, and B3) do not change much in the PSNR and SSIM values of the video when the network changes greatly.

**Figure 8:**
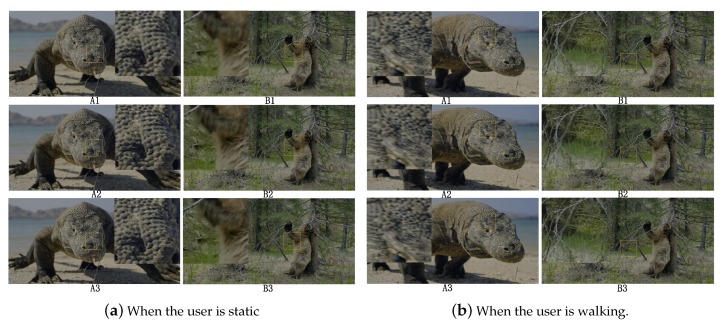
Images in video frame comparison results.

**Figure 9:**
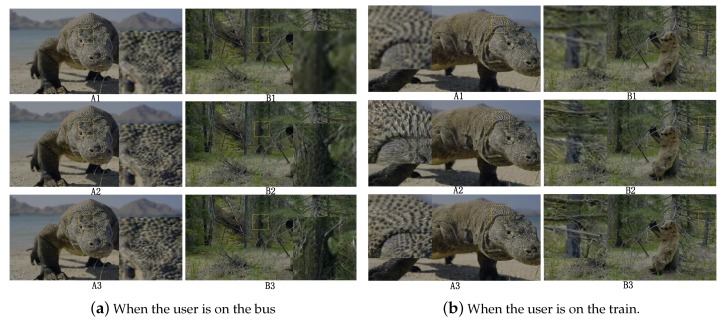
Images in video frame comparison results.

**Figure 10:**
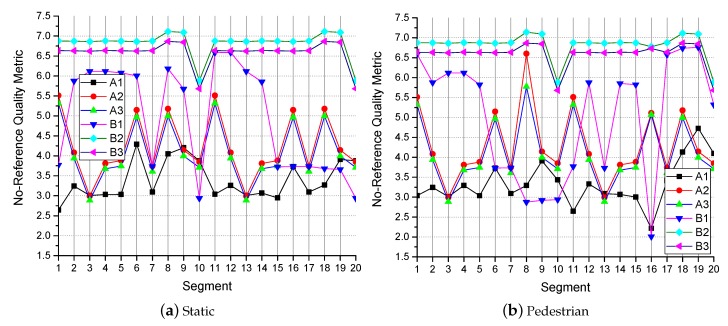

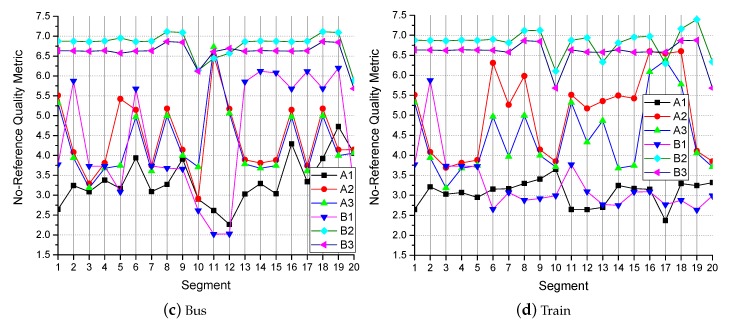
The no-reference quality metric in different network environments.

**Figure 11:**
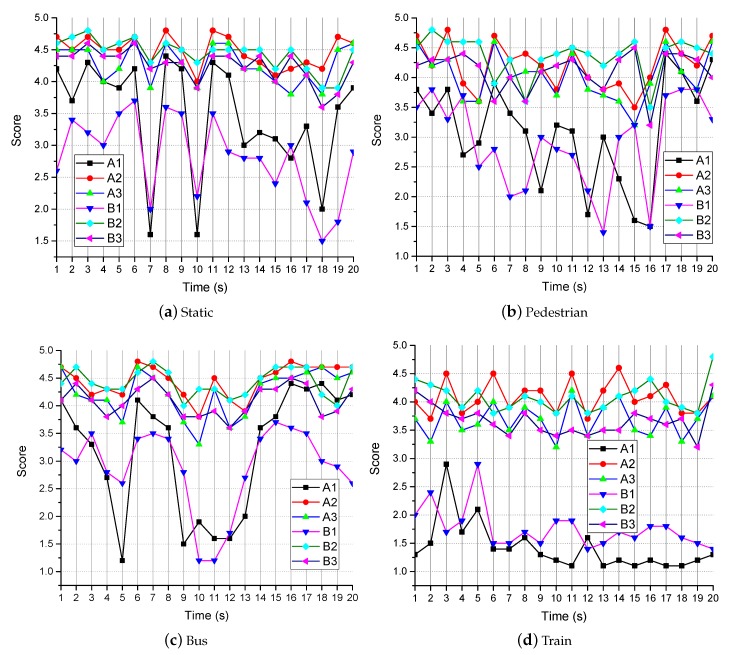
The results of the subjective quality assessment in different network environments.

The images in the video frame comparison results for the transmission methods are shown in Figure 8 and Figure 9. The results of no-reference quality assessment for the transmission methods are shown in Figure 10. The results of subjective quality assessment for the transmission methods are shown in Figure 10 and Figure 11. In Figure 8a,b and Figure 11a,b, the three video transmission methods (A1, A2, A3, B1, B2, and B3) obtain similar video qualities when the network resources are relatively abundant (the network of environment static and pedestrian). In Figure 10a,b, SASRT (A2, A3, B2, and B3) achieves better results than the traditional transmission method (A1 and B1). The reason for the difference in subjective and objective quality evaluation is that the human eye is less sensitive to video than computers. Moreover, as shown in Figure 5a,b, the throughput of SASRT (A2, A3, B2, and B3) is substantially smaller than that of A1 and B1. In Figure 9a,b, Figure 10c,d and Figure 11c,d, the user is in a high-speed state. Users need to switch frequently to connect to different base stations to maintain network connectivity. Therefore, their channel quality will be poor. In this case, the user has a poor video experience using the traditional transmission method (A1 and B1). However, SASRT (A2, A3, B2, and B3) achieves better results under limited network resources. Note that A2 and A3 achieve similar video qualities in Figure 8, Figure 9, Figure 10 and Figure 11 due to the following: The SFTGAN that we used is one of the best super-resolution technologies for images. We lack a super-resolution algorithm based on video transmission. Therefore, A2 spends more network resources obtaining a video quality similar to that of A3. Proposing a transmission-based super-resolution algorithm is the goal of our subsequent research. Because this paper has size constraints, additional experimental results can be obtained from https://drive.google.com/drive/folders/1r4tPuqBgYcKKTigw2s1sMe3C5Z2gXBAX?usp=sharing.

The results of video playback stability are shown in Table 1. The playback stability of SASRT (A1, A2, A3, B1, B2, and B3) is similar for Videos A and B. The playback stability of traditional transmission method (A1) is slightly better than SASRT (A2 and A3) for Video A. However, the playback stability of SASRT (B2 and B3) is much better than the traditional transmission method (B1). We analyzed the reasons as follows: when Video A was taken, the camera moved as the object moved, and the background of the video changed relatively. Video B is a scene taken by a still camera, and the video background is relatively stable. Therefore, the proposed SASRT is more suitable for fixed video sensors to capture video (such as video surveillance scenario [40]), while dynamic video sensors (such as video sensors in the unmanned aerial vehicle) will have poorer performance when capturing video, which requires more optimization.

The semantic recognition location can be calculated by Equation (18). The tradeoff parameters can be specified by the user. In this paper, some suggestions are provided. When the network resources are relatively abundant, our method with the semantic recognition location obtained on the multimedia sensor is recommended. Our method can reduce the network resource cost of the multimedia sensor and enrich the user’s video experience. When the network bandwidth is limited, SASRT with the semantic recognition location obtained by the client is recommended. This can best improve the video quality experienced by users.

## 5. Conclusions

In this paper, SASRT is presented to compress video information redundancy with a long period of time without compromising the user experience over WMSNs. On this basis, a mathematical model for characterizing video quality as affected by certain factors in the proposed SASRT is introduced. Three adaptive strategies are proposed and analyzed individually. We evaluate our performance in a real transmission system. Finally, experiments show that the proposed method can effectively compress video information redundancy over a long period of time and enrich the user experience under limited network resources, thereby improving the utilization of network resources in WMSNs.

There are some limitations to this article. The proposed SASRT is not suitable for dynamic video sensors (such as video sensors in the unmanned aerial vehicle). Dynamic video sensors have a large background change in the video. Super-resolution performance is different in different video scenes. More quality differences exist between video segments, which reduces the quality stability of video playback. Another limitation is that the scene shot by the sensor needs a super-resolution model for this scene. The strategy proposed in this paper requires running deep learning algorithms. Especially user equipment could have power restrictions. Weighing the energy consumption and performance is another issue. The above limitations are also our goal of optimization in the future.

## Figures and Tables

**Table 1:** Playback stability.

	A1	A2	A3	B1	B1	B2
Static	0.804	0.7648	0.7743	0.6996	0.9543	0.9641
Pedestrian	0.7293	0.646	0.7106	0.7363	0.9502	0.9555
Bus	0.7436	0.6765	0.6387	0.7572	0.9506	0.9575
Train	0.8446	0.7923	0.7238	0.5138	0.921	0.9506
